# Green Technology for Pork Loin Wet Curing—Unconventional Use of Cow and Soy Milk Treated with Non-Thermal Atmospheric Plasma

**DOI:** 10.3390/foods11162523

**Published:** 2022-08-21

**Authors:** Monika Marcinkowska-Lesiak, Iwona Wojtasik-Kalinowska, Anna Onopiuk, Adrian Stelmasiak, Agnieszka Wierzbicka, Andrzej Poltorak

**Affiliations:** Department of Technique and Food Development, Institute of Human Nutrition Sciences, Warsaw University of Life Sciences, Nowoursynowska 159c Street, 32, 02-776 Warsaw, Poland

**Keywords:** meat curing, nonthermal plasma, nitrite, milk, soy, powder

## Abstract

This study was conducted to evaluate the possibility of using plasma-activated cow and soy milk powders as a substitute for sodium nitrite for wet curing of pork meat (*m. longissimus thoracis et lumborum*). Pork loin slices were cured for 4 d at refrigerate conditions in four brines: water + salt (NC group), water + salt + sodium nitrite (PC group), water + salt + plasma-activated cow milk powder (B1 group), and water + salt + plasma-activated soy milk powder (B2 group). Importantly, brines from groups PC, B1, and B2 were characterized by the same concentration of NO_2_^−^ ions (200 ppm). Results show that samples from B1 and B2 groups had significantly (*p* < 0.05) higher values of redness, nitrosylhemochrome content, and lower values of thiobarbituric acid reactive substances (TBARS) compared to samples from the NC group. At the same time, the groups cured with alternative curing agents were characterized by lower residual nitrite content with regard to groups cured with NaNO_2_. No significant differences (*p* ≥ 0.05) were found in pH and shear force values among the treatments. Finally, the aroma profile of the samples from groups B1 and B2 was similar to the aroma profile of the samples from the PC group (the aroma differed by a maximum of 1.73% in the case of brine containing plasma-activated cow milk powder) but differed significantly from the NC group (the aroma differed in 97.21%). Due to the higher nitrite depletion in the final product, while maintaining the quality parameters similar to traditionally cured pork loins, both alternative curing agents can be recommended, with a predominance of plasma-treated soy milk.

## 1. Introduction

Plasma is considered to be the fourth state of aggregation. Its production requires an appropriate amount of energy, which is related to the phenomenon of gas ionization and dissociation. The physical properties of the gas change right at the moment when the gaseous state becomes plasma. The most important ones are the appearance of electrical conductivity and the loss of insulation [1,2]. The plasma is called high-temperature plasma when it consists only of ionized particles and free electrons. It is produced by thermonuclear fusion and is a component of stars or a hydrogen bomb. Plasma can also consist of mixtures of ionized and non-ionized molecules, atoms in ground and excited states, and free radicals and electrons. Low-temperature plasma (so-called cold, nonthermal) with a high or very high content of neutral particles occurs in the temperature range from 2000 K to 30,000 K [3,4]. Nonthermal plasma can be additionally divided into low and normal pressure (the first requires a particular booth, and the second, in turn, can be incorporated into continuous processes in technological lines) [1].

So far, research on cold plasma has focused mainly on its use in microbial decontamination of foods [5,6,7]. It is due to the synergistic effect of plasma-generated reactive species (RS) and UV radiation, which cause oxidative changes in the lipids and proteins of the plasmalemma and damage nucleic acids, thus leading to the destruction of microbial cells [8]. More and more research [9,10,11] is highlighting the fact that the reactive forms produced by low-temperature plasma may decrease food pH or modify the functional properties of food ingredients, such as polysaccharides or proteins, influencing, e.g., their viscosity, solubility, or water absorption. The extent of the observed changes will depend on the composition of the product and the properties of the plasma itself, such as treatment time, the type of gas, and applied pressure or power supply [12]. Nevertheless, the undoubted advantage of this method is that it does not raise the temperature of the product, has a limited impact on its organoleptic properties at lower power intensities/shorter treatment time, and is environmentally friendly at the same time [11].

Recently, due to the current trend of reducing the list of synthetic additives in meat products, more and more alternative curing methods are being developed, including those using nonthermal atmospheric plasma for this purpose [13]. So far, curing is usually considered to be the addition of salt (NaCl) with sodium nitrite (NaNO_2_) or potassium nitrite (KNO_2_) to meat during its processing [14]. As a result, nitrite (NO_2_^−^) accepts hydrogen ions (H^+^) and generates nitrous acid (HNO_2_), which gradually decomposes into dinitrogen trioxide (N_2_O_3_) and a water molecule. Dinitrogen trioxide (N_2_O_3_) can, in turn, dissociate into nitric oxide (NO) and nitrogen dioxide (NO_2_). Due to nitrogen’s easily changeable oxidation status, many reactions of nitrite derivatives with meat ingredients also take place. This leads to the inhibition of microbial growth (through reactions of nitrites with cations present in bacterial cells) and oxidation processes (through oxidation of nitrites to nitrates as well as their reaction with iron present in heme), as well as to the formation of characteristic color (through conversion of nitrosylmyoglobin after heat treatment to a stable, pink nitrosylhemochrome) and taste (through nitrites reactions with proteins and lipids) of cured meat products [15,16].

Curing using nonthermal atmospheric plasma is possible because of reactive forms of nitrogen (RNS),which react with water molecules to form nitrites. Nevertheless, to obtain an adequate level of nitrite, the water-containing matrices exposed to the plasma should have a high pH and be able to maintain it at a relatively constant level for as long as possible. This is because the overproduction of hydrogen ions by plasma action could lead to the degradation of the nitrite produced [17]. In the existing literature, it has been observed that research on the use of cold plasma as an alternative curing method focuses mainly on the methods involving the addition of plasma-activated matrices like water with chemical buffers [18,19,20], plant protein preparations solutions [21], vegetable powders [22,23], or even milk powder [24] to the meat during mixing with fat, cooled water/ice, and other functional additives. Nevertheless, apart from dry, wet curing methods can also be distinguished. In those methods, the meat can be immersed in brine containing a given concentration of nitrite or injected with brine solution to support brine penetration [25,26]. 

In immersion curing, we are dealing with an osmosis process because it increases the solute concentration in the meat and hence decreases its water potential. The higher viscosity of the brine can cause the creation of a barrier that hinders the mass exchange between the raw material and the solution [27,28,29]. In our previous studies [21,24] we demonstrated the potential of using plasma-activated soy and milk proteins for dry curing methods, so in this study we concentrated on their application in the immersion curing method. Therefore, the main objective of the present study was to evaluate the application of cow and soy milk treated with nonthermal atmospheric plasma in the immersion curing of pork loin. All treatments were assessed for their residual nitrite content and we chose quality parameters to confirm the applicability of this green technology to meat curing.

## 2. Materials and Methods

### 2.1. Materials and Reagents

The pork loins (*m. longissimus thoracis et lumborum*) weighing 2.85 ± 0.15 kg (n = 9) were obtained from a local processor (Wierzejki, J. M. Zdanowscy JSC, Płudy, Poland). Cow milk (ultra-heat-treated product, 2 g of fat per 100 mL, 3.3 g of protein per 100 mL, MLEKPOL, Ltd., Kolno, Poland) and soy milk (ultra-heat-treated product, 2 g of fat per 100 mL, 3 g of protein per 100 mL, MLEKPOL, Ltd., Poland) were purchased locally. The reagents were collected from Sigma-Aldrich Co., Ltd. (St. Louis, MO, USA).

### 2.2. Preparation of Curing Agents

**Figure 1:**
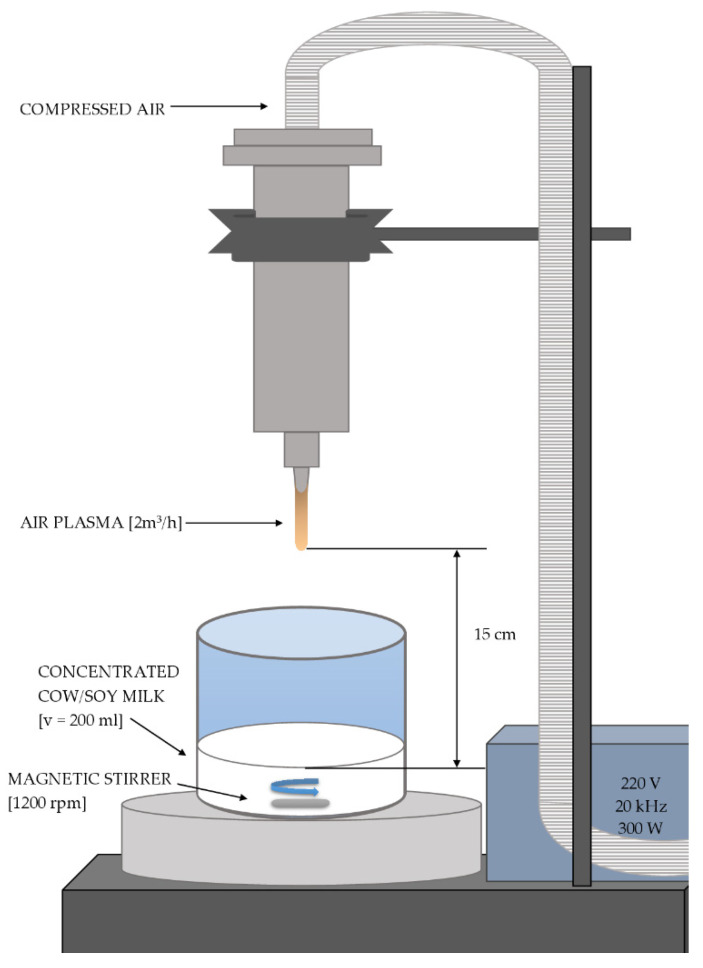
Scheme of cow/soy milk treatment with plasma jet system.

To manufacture alternative curing agents, cow and soy milk were first concentrated to 50% of their original volume using a vacuum rotary evaporator (Rotavapor R-3 Büchi, Essen, Germany) at 70 mb pressure and 55 °C. Then, 200 mL aliquots were exposed to a nonthermal plasma source at atmospheric pressure (Diener electronic GmbH & Co. KG, Ebhausen, Germany), as shown in Figure 1. 

Plasma treatment was carried out until the pH of the samples was 6.00 ± 0.02. Then, all portions were lyophilized using a freeze dryer (ChristAlpha 2-4 LSC plus model, Osterode am Harz, Germany) according to the method described in the study by Marcinkowska-Lesiak et al. [24]. Finally, lyophilizates were ground using a mortar and passed through a vibrating sieve (RetschGmbK, Hann, Germany) with a mesh size of 425 µm. The residual nitrite content in obtained powders was analyzed according to the method of Lee et al. [30] (described in Section 2.4). The above process was performed in triplicate. The mean nitrite content in the plasma-treated cow and soy milk powders was 1317.78 ± 138.17 mg/kg and 2642.55 ± 221.66 mg/kg, respectively. Before use, both curing agents were packaged under vacuum in polyamide/polyethylene pouches and stored at −60 °C.

### 2.3. Processing of Cured Pork Loins

Then, 24 h post mortem, a total of nine left carcass loins (*m*. *Longissimus thoracis et lumborum*) weighing 2.85 ± 0.15 kg were separated from randomly selected carcasses. None of them were recognized as Pale Soft Exudative (PSE) or Dark Firm Dry (DFD). Pork loins were transported in cold conditions (2 ± 1 °C) to the Department of Technique and Food Development (Warsaw University of Life Sciences, Poland). At the 48th h post mortem, after trimming connective tissue and excessive fat, 4 slices with a thickness of 2.54 cm were taken from the middle of each loin and randomly allocated to 4 treatments that differed in the brine used in the curing process: NC (water—75% by the weight of the meat and NaCl—6% by the weight of the meat, nitrite free), PC (water—75% by the weight of the meat, NaCl—6% by the weight of the meat, NaNO_3_—0.03% by the weight of the meat), B1(water—75% by the weight of the meat, NaCl—6% by the weight of the meat, plasma-activated cow milk powder–15% by the weight of the meat), and B2 (water—75% by the weight of the meat, NaCl—6% by the weight of the meat, plasma-activated soy milk powder—7.5% by the weight of the meat). The proportions of the individual components of the brine were converted so that in groups PC, B1, and B2, there was the same content of NO_2_^−^ ions (200 mg per kg of meat). In each group, the above process was carried out separately for each pork loin slice in 0.5 L glass beakers. After soaking slices in prepared solutions, they were cured in refrigerated conditions (4 ± 1 °C) for 4 d. Finally, pork loin slices were weighed and packed in polyamide/polyethylene bags, heated in a water bath (75 °C) for 30 min, and cooled at room temperature for 1 h. A total of 36 pork slices (9 slices/treatment) were used.

### 2.4. Physicochemical Analyzes

The curing yield was calculated based on the difference in weight of pork loin slices before and after the curing process. Meanwhile, the cooking loss was calculated through the difference in weight of the samples before and after heating in the water bath. Both results were converted to percentages.

The pH of the plasma-treated cow and soy milk concentrates was analyzed every 5 min with a FiveEasy^TM^ F20 meter (Metler Toledo LLC, Columbus, OH, USA) until the pH was 6. The pH values of the pork loin slices were measured after curing and heat treatment with a food pH meter with penetration probe (model 205, Testo Inc., Lenzkirch, Germany). Before the measurements, the calibration was performed with standardized buffers at room temperature. A triple measurement was performed for 1 sample.

The color of the raw cured samples was measured on the outer surface, while cooked samples were sliced across, and their internal surface color was determined immediately after cutting. Measurements were made with a Minolta chromometer (CR-400, Konica Minolta Inc., Tokyo, Japan) with a standardized light source (D65 illuminator), viewing angle 2°, and spot diameter 8 mm. Before measurements, the equipment was calibrated with the white standard calibration plate (L* = 98.45, a* = −0.10, b* = −0.13). L* (lightness), a* (redness), and b* (yellowness) values were determined for all samples. From each sample, 3 measurements were made from random places. Based on the parameters L*, a*, and b*, the chroma (C*) of pork loins from each treatment and the total color difference (ΔE) relative to the NC group were calculated as follows:

(1) $$C^{*}=\sqrt{a^{*2}+b^{*2}}$$

(2) $${\Delta E}^{*}=\sqrt{{\Delta L}^{*2}+{\Delta a}^{*2}+{\Delta b}^{*2}}$$

Nitrite content in obtained powders as well as in cooked loins was measured with the modified method of Lee et al. [30]. Then, 10 mL of an appropriately diluted solution of the obtained powders or a 10 g sample of the loin was placed in a 250 mL flask and mixed thoroughly after adding hot water (80 °C). Next, 10 mL of sodium hydroxide (0.5 M) was added and mixed, and the same was done with 10 mL of zinc sulfate (12%). After heating in a shaking water bath (80 °C) for 20 min, cooling for 10 min in tap water, and thoroughly mixing with 2 mL of 10% ammonium acetate, the pH of which had previously been adjusted to 9.1 with ammonia water, all solutions were filtered using Whatman no. 1 paper. Then, 1 mL of 30 mM sulfanilamide in acid solution (HCl:H_2_O = 1:1, *v*/*v*) and 1 mL of 5 mM N-(1-naphthyl) ethylenediamine dihydrochloride were added to 20 mL of each filtrate and finally diluted with deionized water to 25 mL. The absorbances of all samples were determined after 20 min at 540 nm using a cuvette spectrophotometer (SparkTM 10 M, Tecan Group, Männedorf, Switzerland).The residual nitrite content was calculated from a calibration curve based on working solutions of sodium nitrite. For each sample, 3 measurements were made.

Also, the content of nitrosylhemochrome in pork loins was determined by a slightly modified method by Lee et al. [30]. First, samples weighing 5 g were homogenized with 1.5 mL of distilled deionized water and 20 mL of acetone using Ultra Turrax homogenizer (T18 basic, IKA Werke, Staufen, Germany). After the solutions were stored in the dark for 15 min and filtered through Whatman no. 1 filter paper, their absorbance at 540 nm was measured with a cuvette spectrophotometer (Spark^TM^ 10M, Tecan Group, Mannedorf, Switzerland). The concentration of nitrosylhemochrome (ppm) in all samples was calculated by multiplying their absorbance by 290. Next, samples of the same weight (5 g) were homogenized with 1 mL of distilled deionized water, 0.5 mL of HCl, and 20 mL of acetone using Ultra Turrax homogenizer (T18 basic, IKA Werke, Staufen, Germany). After the solutions were stored in the dark at 4 °C for 1 h and filtered through Whatman no. 1 filter paper, their absorbance at 640 nm was measured with a cuvette spectrophotometer (Spark^TM^ 10M, Tecan Group, Mannedorf, Switzerland). The total pigment concentration (ppm) in all samples was calculated by multiplying their absorbance by 680. The nitrosylhemochrome content (%) of pork loins was calculated by dividing the nitrosylhemochrome concentration by the total pigment concentration and multiplying the result by 100. Three measurements were made for each sample.

The lipid oxidation of pork loins was assessed by the value of substances reactive with 2-thiobarbituric acid (TBARS) according to the method of Pikul et al. [31] with modifications described in Marcinkowska-Lesiak et al. [21,24]. The TBARS values of the samples were calculated using their absorbance measured at 532 nm using a cuvette spectrophotometer (SparkTM 10 M, Tecan Group, Mannedorf, Switzerland) and expressed as mg malondialdehyde equivalent/kg sample. The measurements were made in triplicate for each sample.

Warner-Bratzler shear force (WBSF) measurements of cooked loins after being cooled down to 4 °C were performed using an Instron universal testing machine (Model 5965, Instron Co., Canton, MA, USA). Three cylindrical samples (diameter of 1.27 cm) from each pork loin slice were cut in a direction parallel to the longitudinal orientation of the muscle fibers. Then cores were sheared using a V-shaped steel knife perpendicular to their long axis. The capacity of the measuring head of the machine with a crosshead speed of 200 mm/min was 500 N. WBSF (N) was defined as the peak force of the curve.

### 2.5. Aroma Profile

The aroma profile of pork loins was analyzed with an electronic nose (Heracles II, Alpha M.O.S., Toulouse, France). For this purpose, samples of a weight of 2 g were placed in a 20 mL headspace vial, capped with Teflon-faced silicon rubber. Then samples were treated according to the method described by Wojtasik-Kalinowska et al. [32]. Ultimately, specific volatile compounds were identified based on AroChemBase (Alpha MOS Co., Toulouse, France).

### 2.6. Statistical Analysis

Statistical analysis was performed with the General Linear Model (Statistica 13.3, StatSoft Inc., Tulsa, OK, USA). Treatment was assigned as a fixed effect and carcass as a random effect. Since the carcass had no effect on the results, the influence of curing methods on pork loin physicochemical traits was investigated using a one-way analysis of variance (ANOVA), and significant differences (*p* < 0.05) between the samples were tested according to Tukey’s multiple range test at the significant level of *p* < 0.05. The main component analysis (Alpha Soft version 8.0, Toulouse, France) was used for the fragrance profile data. The obtained results were presented as mean values and standard errors (SE).

## 3. Results and Discussion

### 3.1. Curing Yield and Cooking Loss

**Figure 2:**
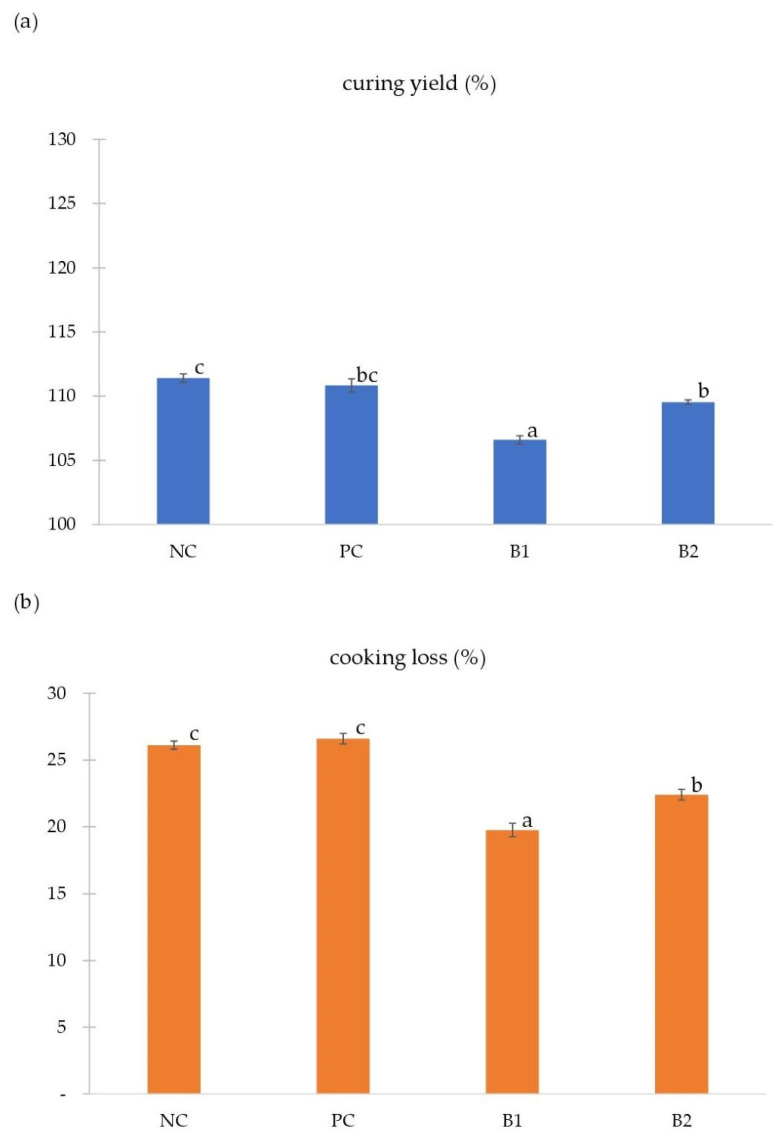
The effect of alternative nitrite sources on curing yield (**a**) and cooking loss (**b**) of cured pork loins (mean ± SE); NC—negative control (pork loins soaked in nitrite-free brine); PC—positive control (pork loins soaked in brine containing sodium nitrite); B1—pork loins soaked in brine containing plasma-activated powder of cow milk; B2—pork loins soaked in brine containing plasma-activated powder of soy milk; a–c—the mean values with different letters differ statistically significantly (*p* < 0.05).

Figure 2 includes curing yield (a) and cooking loss (b) values of analyzed pork loins. Both parameters were influenced by the curing method (*p* < 0.05). There were no significant differences (*p* ≥ 0.05) in the values of curing yield (CY) and cooking loss (CL) among NC and PC groups, which was consistent with the studies of Kim et al. [33,34], who analyzed nitrites from chard and spinach in terms of quality parameters of cured pork loins. Nevertheless, the values of both parameters significantly decreased (*p* < 0.05) in samples from groups B1 and B2 compared to groups NC and PC. Contrary to our results, in the mentioned studies no differences were either found in the curing yield and cooking loss values of groups cured in brines containing alternative or conventional sources of nitrites [33,34]. In the case of wet curing, the driving force for the penetration of water and other substances into or from the interior of complex cellular structure is the difference in chemical potential between meat and its surrounding brine [25]. Brine ingredients migrate into the meat and the water from the inside of the meat moves into the brine. The process stops when the salt pressure equalizes on both sides [28]. Nevertheless, after penetrating into the meat fibers, salt causes them to swell and increases the ability to absorb and retain more water from the brine. This gain will depend on salt concentration, curing method, and curing time, among other factors. In all the analyzed treatments, the addition of NaCl to the brines was the same. The differences in CY values observed in our work may thus result from different properties characterizing brine’s internal friction. The penetration of water, salt, and nitrite decreases when the viscosity of brine increases, which is related to the presence of protein and fat, among others [35]. On the other hand, the mass transfer may also be hindered because of the creation of a barrier by high molecular weight components on the outer layers of raw material [36]. Therefore, pork slices from groups B1 and B2 were characterized by lower values of curing yield (*p* < 0.05, Figure 2a) than the samples from groups NC and PC. In turn, the loss of mass after the heat treatment of meat results from the greater mobility of water caused by protein denaturation [37]. Importantly, high cooking loss of pork may give the expectation of a less-optimal eating quality [38]. In our study, the lowest average value of the cooking loss was obtained in group B1 (*p* < 0.05, Figure 2b). Also, group B2 was characterized by lower values of the CL parameter compared to the NC and PC groups (*p* < 0.05). It can be explained by the fact that heat in groups B1 and B2 destroys and makes proteins on the pork slices’ surface stick together into an aggregated [39] form that hinders water evaporation.

### 3.2. pH and Color Parameters

According to Luo et al. [40], plasma-treated water may lower the pH of cured meat because of the presence of brine NO_x_ molecules and H_2_O dissociation. The results of our study did not show a significant effect of brine (Table 1, *p* ≥ 0.05) on the pH values of cured pork loins, which were, on average, 5.55 and 5.80 for slices after the curing and heat treatment, respectively. In addition, our earlier studies showed that pork sausages with both plasma-treated soy solution and plasma-treated milk powder had a higher pH than sausages with NaNO_2_ and sausages without nitrites [21,24]. However, in mentioned works [21,24], these alternative curing agents were added directly to the raw material during the mixing stage. In contrast, in this study, they were parts of the brines, which could lead to their limited uptake.

**Figure 3:**
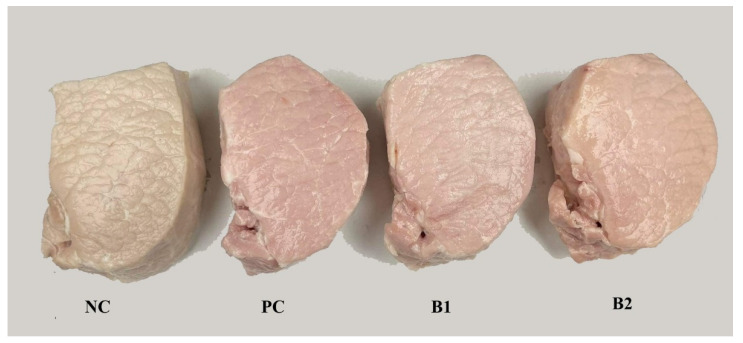
Cured pork loins–top view; NC—negative control (pork loins soaked in nitrite-free brine); PC—positive control (pork loins soaked in brine containing sodium nitrite); B1—pork loins soaked in brine containing plasma-activated powder of cow milk; B2—pork loins soaked in brine containing plasma-activated powder of soy milk.

Contrary to the pH value, all the color parameters (L*, a*, b*) of the outer surface of raw pork loins and the inner surface of cooked pork loins were influenced by the curing method (*p* < 0.05; Table 1). After the curing process, an increase in lightness (L* values) and redness (a* values) was observed for the PC, B1, and B2 groups compared to the group without nitrite (*p* < 0.05). Additionally, pork loins cured in brines containing plasma-activated cow and soy milk powders were characterized by lower values of a* and higher values of b* parameters compared to samples cured with a mixture of salt and sodium nitrite (*p* < 0.05). These differences in redness may be related to the different content of nitrosomyoglobin, characterized by a pinkish-red color in groups PC, B1, and B2 [41]. Additionally, the yellowish color of the surface of the soaked samples from groups B1 and B2 was probably related to the pigments resulting from the reaction of reactive forms of plasma with cow or soy milk [42]. After heat treatment and cooling (Figure 3), pork loins were sliced across, and their internal surface’s color was determined.

Table 1 also shows the changes in color parameters of cooked samples. The curing process significantly decreased (*p* < 0.05) the lightness of samples compared to those from the control group NC. Nevertheless, the color of brines may be the reason for different values of L* parameter of pork loins from groups B1 and B2 compared to samples soaked with water and a mixture of salt and sodium nitrite. Pork loins cured with plasma-activated cow and soy milk powders were characterized by significantly higher lightness than the PC group (*p* < 0.05). The cured samples were also redder than those from the NC group, with the highest values of a* parameter recorded for the pork loins from the PC group. The higher values of redness in groups PC, B1, and B2 are the result of the formation of nitrosyl hemochrome from the degradation of nitrosomyoglobin during heat treatment [40]. Samples cured with sodium nitrite also had a less-yellow color than pork loins from groups NC, B1, and B2 (*p* < 0.05). In slices soaked in brines with plasma-activated cow and soy milk powders, the b* parameter did not differ after heat treatment from nitrite-free samples. Regarding groups B1 and B2, the yellow color component of cooked pork loin may come from the pigments of plasma-activated cow and soy milk [42].

Based on the values of L, a, and b, the parameter C* (chroma) and the total color difference (E) were also calculated according to the formulas given in Section 2.4. The mean values of the above parameters in groups PC, B1, and B2 differed significantly (*p* <0.05, Table 1) from the values in the NC group, both after curing and after heat treatment. In the case of cooked slices, the obtained ΔE values were above 3.5, which indicates apparent color differences [43] among the groups containing nitrites and the nitrite-free group. Additionally, the NC group was characterized by the lowest values of the C* parameter (*p* < 0.05), which indicates that the pork loins of this group were the palest [44]. In turn, the highest values of chroma were obtained in group B2, which can be attributed to the penetration of nitrites from the brine into the meat and pigments of plasma-activated soy milk.

### 3.3. Residual Nitrite and Nitrosylhemochrome Content 

**Figure 4:**
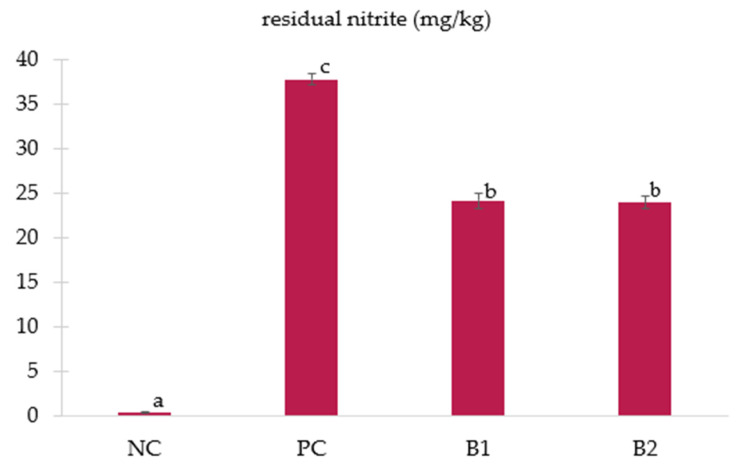
The effect of alternative nitrite sources on residual nitrite (mg/kg) of cured pork loin (mean ± SE); NC—negative control (pork loins soaked in nitrite-free brine); PC—positive control (pork loins soaked in brine containing sodium nitrite); B1—pork loins soaked in brine containing plasma-activated powder of cow milk; B2—pork loins soaked in brine containing plasma-activated powder of soy milk; B2—pork slices soaked with brine based on plasma-activated powder of soy milk; a–c—the mean values with different letters differ statistically significantly (*p* < 0.05).

Too-low residual nitrite concentrations have potentially harmful effects due to their inability to destroy meat pathogens. On the other hand, high concentrations of NO_2_ can lead to the formation of nitrosamines in processed meat. It is assumed that for immersion muscle, maximum ingoing nitrite concentrations should be 200 ppm [45]. As expected, cured samples were characterized by higher nitrite levels (*p* < 0.05, Figure 4) than samples soaked in water with salt (NC–0.43 ± 0.10 mg/kg). In addition, the content of residual nitrite in cured pork loins differed significantly (*p* < 0.05) depending on the source of nitrite used.

As there were no differences in the pH values (*p* ≥ 0.05; Table 1) among the PC, B1, and B2 groups, and the brines used in these groups did not differ in terms of used antioxidants, authors assumed based on literature [33,34] that nitrite depletion rates in these groups might be similar. Although none of the cured samples crossed the Codex Alimentarius limit of 80 ppm of nitrite for heat-treated processed meat, poultry, and game products in whole pieces or cuts [46], the average content of residual nitrite in samples from the PC group (37.74 ± 0.66 mg/kg) was significantly higher (*p* < 0.05) than in samples from groups B1 (24.13 ± 0.83 mg/kg) and B2 (23.91 ± 0.72 mg/kg) even though the same amount of nitrite ions was added to the brines. Additionally, pork loin slices soaked in brine containing plasma-activated cow milk or plasma-activated soy milk did not differ in residual nitrite values (*p* ≥ 0.05). In general, in groups with alternative nitrite sources, about 36–37% fewer residual nitrites were noted in relation to the PC group. This is beneficial because, taking into account the maximum acceptable daily consumption (ADI) of nitrite at the level 0.07 mg/kg/day, a healthy adult person (weighing about 60 kg) could consume even up to 180 g of the product from the B1 or B2 and only 110 g of the product from the PC group [45]. The differences in the obtained nitrite contents in groups B1 and B2 in relation to the PC group may, therefore, result from the hindered transport of nitrites to the meat caused by a higher viscosity as well as a higher molar mass of brines used in groups B1 and B2. These assumptions are confirmed, for example, by the results concerning the curing yield (Figure 2a). Nevertheless, Yong et al. [18] also demonstrated a reduction in residual nitrite content in samples of loin ham cured with plasma-treated water compared to samples cured with sodium nitrite. Therefore, more research is needed to elucidate the reasons behind the lower residual nitrite content in soaked meat in various brines containing alternative nitrite sources.

Nitrosomyoglobin, after denaturation by heat treatment, transforms into a stable red pigment—nitrosylhemochrome [47]. The redness of cured pork loins in the above study was, therefore, influenced by the content of nitrosylhemochrome. As expected, compared with the NC group (pork loins soaked with brine not containing nitrite source), other groups (PC, B1, B2) were characterized by significantly higher values of nitrosylhemochrome (*p* < 0.05; Table 2). In addition, similarly to the residual nitrite contents, our results showed that samples soaked in brine containing sodium nitrite (PC group) had a higher content of nitrosylhemochrome (*p* < 0.05) than the samples from groups with plasma-activated cow and soy milk powders. The lowest average content of nitrosoheme pigment among samples was soaked in brines containing sources of nitrites obtained in group B1. Our previous studies have shown that compared to sausages with sodium nitrite, it is possible to achieve similar [21] or even higher [24] levels of nitrosylhemochrome using a plasma-activated soybean solution or plasma-activated milk powder when these alternative curing agents are added to the meat ingredients during mixing. Nevertheless, the results obtained in this study indicate that despite the formation of a pigment characteristic of cured meat in the case of using alternative sources of nitrites for wet curing, the obtained values were about 9% and 18% (respectively for groups B2 and B1) lower than the PC group. Importantly, these differences did not cause a drastic decrease in the redness of samples from groups B1 and B2 compared to the PC group (Table 1).

### 3.4. Thiobarbituric Acid Reactive Substances and Warner-Bratzler Shear Force

Thermal treatment affected lipid oxidation in the NC group the most (Table 2). The average value in the above group was similar to values obtained by Karwowska et al. [48] and showed that pork loins were not yet rancid (<1.5 mg MDA/kg) [49]. As expected, curing determined a lower initial oxidation of the pork loins. It is because nitrite reacting with iron ions of myoglobin reduces free iron and thus delays lipid oxidation [20]. According to the literature, a nitrite dose of 20–50 mg/kg of the product is sufficient to obtain the desired antioxidant effect [45]. The ingoing nitrite contents in groups PC, B1, and B2 were not below the above-mentioned minimum range. Therefore, the TBARS values in these samples were probably significantly lower than those soaked only with water and salt (*p* < 0.05; Table 2). Nevertheless, pork loin slices cured in brine containing sodium nitrite showed the lowest degree of oxidation (0.24 mg MDA/kg), monitored by markers of secondary lipid oxidation products, compared to pork loin slices cured in brines with plasma-activated cow (B1) and soybean powders (B2). The samples from groups B1 and B2 did not differ statistically significantly (*p* ≥ 0.05) in terms of the discussed parameter, reaching values in the range of 0.32 to 0.34 mg MDA/kg. Thus, our results are in line with previous studies [33,34,40], which have shown that alternative sources of nitrite (both derived from plasma-treated raw materials as well as from plants as a result of nitrate conversion) may be sufficient to maintain the oxidative stability of wet-cured meat products.

Consumer satisfaction with meat products is related to tenderness, which is considered the most essential feature of palatability [50]. All the analyzed treatments were characterized by similar shear force values from 19.58 to 20.79 N (*p* ≥ 0.05, Table 2). Considering the above, the presence of alternative sources of nitrite did not deteriorate the tenderness of the cooked pork loins. Contrary to our studies, Kim et al. [33] showed differences in WBSF values depending on the type of nitrite source. According to the authors, the reason for the higher shear force values of pork chops soaked in a solution of fermented beets may be related to the lower pH of the samples. In our study, however, no differences in the pH of the pork chops were found.

### 3.5. Volatile Compounds

**Figure 5:**
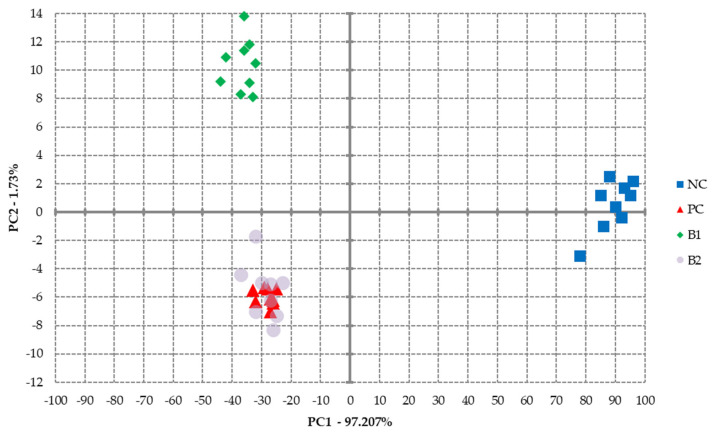
PCA analysis of cooked pork loins; NC—negative control (pork loins soaked in nitrite-free brine); PC—positive control (pork loins soaked in brine containing sodium nitrite); B1—pork loins soaked in brine containing plasma-activated powder of cow milk; B2—pork loins soaked in brine containing plasma-activated powder of soy milk.

The aroma of cured meat is mainly due to the reaction of nitric oxide or nitrite with proteins and fats; however, according to Flora et al. [51], the use of nitrites cannot be attributed to the formation of a specific cured aroma compound but should constitute a balance between aldehyde compounds and sulfur-a key meat odor compounds. In our study, the principal component analysis (PCA) was used to identify the most important elements of variability in the aroma of pork loins soaked in different brines (Figure 5, Table 3). Figure 5 represents the classification of scent profiles with regard to the treatment they belonged to (NC, PC, B1, and B2). Samples are represented in a two-dimensional plane with respect to the selected components: PC1 and PC2. The values of 1.73% data variance explained by the horizontal axis and 97.21% intercepted by the vertical axis explain the differences among samples along the axis, indicating that they were much more significant along the abscissa axis than along the ordinate axis. Using different sources of nitrites resulted in changes in the aroma profile of cooked pork loins of the slices soaked only in water and salt (NC group). Group B2 was the most similar to the PC group in terms of aroma. PCA analysis shows that the aroma of B1 group also differed from the PC group by only 1.73%. Importantly, in the case of wet curing, the observed differences in the aroma profile of the cooked pork loin (PC, B1, B2) were smaller compared to the differences in the aroma profile of sausages containing sodium nitrite or plasma-activated soybean solution [21], as well as sodium nitrite or plasma-activated milk powder [24].

After analyzing all the groups, a total of 42 volatile compounds were identified, of which 21 volatile compounds were detected in the NC group, 17 in the PC group, 23 in the B1 group, and 12 in the B2 group (Table 3). Among them, the most numerous were: alcohols (9), aldehydes (10), terpenes (6), and esters (5). Although alcohols or aldehydes have high odor thresholds compared to other compounds, they can be the key aroma components if their concentrations exceed the odor detection threshold [51]. Accordingly, the presence of 2-methylpentanal and hexanoic acid, which impart a cheese flavor in pork loin slices soaked in brine containing plasma-activated powdered milk, may have contributed to the observed differences in the aroma profile among the PC and B1 groups (Figure 5). Importantly, the presence of both benzaldehyde as a decomposition product of alphalinic acid and pyrazine as a product of the Maillard reaction in the presence of lipid oxidation products in the NC group proves their higher degree of oxidation compared to the samples from groups PC, B1, and B2 [40,51], which is also confirmed by the TBARS values in this group (Table 2).

## 4. Conclusions

Currently, there are more and more possibilities for using nonthermal plasma in various branches of the food industry. Due to the growing interest in alternative sources of nitrites, the above study was focused on the possibility of using plasma to produce nitrites in cow and soy milk, which then, in the form of powders (which would facilitate their dosing and storage), would be used in wet curing of meat. 

According to our results, plasma-activated powders of cow and soy milk seem to be an attractive alternative to sodium nitrite in wet curing due to the higher nitrite depletion in the final product while maintaining its desired quality parameters. Both nonconventional curing agents positively affected the redness, nitrosylhemochrome content, and oxidative stability of the final product compared to the slices soaked in water with salt (NC group). Additionally, samples from groups B1 and B2, characterized by lower values of the curing yield and, at the same time, lower cooking loss compared to the PC group, ultimately did not differ from this group in terms of the pH values and tenderness of the final product. Interestingly, group B1 was more similar to the PC group in terms of aroma profile, possibly due to the perceptible cheese flavor in group B2. 

To summarize, as low pressure is not required, the above technology is easy to implement in practice. Additionally, despite the slightly higher costs of the final product, wet curing with plasma-activated cow and soy milk powders is environmentally friendly because of zero residue formation, as in the case of sodium nitrite production. Nevertheless, despite the potential of the proposed curing methods, future research should include a careful analysis of all compounds that may arise in meat products containing plasma-activated cow milk and soybean milk powders.

## Figures and Tables

**Table 1:** The effect of alternative nitrite sources on pH and color parameters (mean ± SE) of cured pork loin (n = 9).

GROUP ^1,2^	pH	L* (−)	a* (−)	b* (−)	C*	ΔE
	raw					
NC	5.54 ± 0.02	53.10 ^b^ ± 0.26	1.99 ^a^ ± 0.11	−0.21 ^a^ ± 0.08	2.04 ^a^ ± 0.11	-
PC	5.53 ± 0.02	52.15 ^b^ ± 0.33	3.27 ^b^ ± 0.10	−0.52 ^a^ ± 0.11	3.37 ^b^ ± 0.10	2.50 ^a^ ± 0.16
B1	5.55 ± 0.01	48.96 ^a^ ± 0.31	3.81 ^c^ ± 0.11	1.40 ^b^ ± 0.14	4.11 ^c^ ± 0.12	5.09 ^c^ ± 0.31
B2	5.56 ± 0.01	52.55 ^b^ ± 0.31	3.99 ^c^ ± 0.10	2.42 ^b^ ± 0.15	4.72 ^d^ ± 0.12	3.84 ^b^ ± 0.17
	cooked					
NC	5.78 ± 0.01	76.98 ^d^ ± 0.09	6.51 ^a^ ± 0.04	5.70 ^b^ ± 0.06	8.66 ^a^ ± 0.04	-
PC	5.82 ± 0.01	70.43 ^a^ ± 0.28	11.57 ^c^ ± 0.14	5.10 ^a^ ± 0.05	12.24 ^b^ ± 0.14	6.30 ^a^ ± 0.26
B1	5.79 ± 0.01	72.79 ^c^ ± 0.23	11.12 ^b^ ± 0.15	5.73 ^b^ ± 0.08	12.53 ^b^ ± 0.13	8.33 ^b^ ± 0.31
B2	5.81 ± 0.02	71.93 ^b^ ± 0.23	11.14 ^b^ ± 0.12	5.87 ^b^ ± 0.07	12.98 ^c^ ± 0.12	6.92 ^a^ ± 0.23

^1^ NC—negative control (pork loins soaked in nitrite-free brine); PC—positive control (pork loins soaked in brine containing sodium nitrite); B1—pork loins soaked in brine containing plasma-activated powder of cow milk; B2—pork loins soaked in brine containing plasma-activated powder of soy milk; ^2^ a–c—the mean values within columns with different superscript letters differ statistically significantly (*p* < 0.05).

**Table 2:** The effect of alternative nitrite sources on nitrosylhemochrome content, thiobarbituric acid reactive substances values, and Warner-Bratzler shear force (mean ± SE) of cured pork loin (n = 9).

GROUP ^1,2^	Nitrosylhemochrome Content (%)	TBARS ^3^ (mg MDA/kg)	WBSF ^4^ (N)
NC	13.36 ^a^ ± 0.72	1.19 ^c^ ± 0.04	20.79 ± 0.84
PC	42.59 ^d^ ± 0.75	0.24 ^a^ ± 0.01	20.69 ± 0.56
B1	34.85 ^b^ ± 0.83	0.34 ^b^ ± 0.01	19.58 ± 0.27
B2	38.67 ^c^ ± 0.79	0.32 ^b^ ± 0.01	20.12 ± 0.26

^1^ NC—negative control (pork loins soaked in nitrite-free brine); PC—positive control (pork loins soaked in brine containing sodium nitrite); B1—pork loins soaked in brine containing plasma-activated powder of cow milk; B2—pork loins soaked in brine containing plasma-activated powder of soy milk; ^2^ a–c—the mean values within columns with different superscript letters differ statistically significantly (*p* < 0.05); ^3^ TBARS—Thiobarbituric acid reactive substances; ^4^ WBSF—Warner-Bratzler shear force.

**Table 3:** Volatile compounds’ characteristic of pork loins cured with alternative nitrite sources.

Compounds	DB5 ^1^	Sensory Descriptors	NC ^2^	PC	B1	B2
alcohols						
Ethanol	450	alcoholic		+ ^3^	+	+
Methanol	451	cabbage	+			
1-propanol	519	alcoholic			+	+
2-mercaptoethanol	562	strong; sulfurous	+			
1-propanol, 2-methyl	636	alcoholic			+	
Propylenglycol	733	alcoholic		+		
2-furanmethanol	843	alcoholic; bread	+	+		
(Z)-2-hexene-1-ol	869				+	
Trans-Carveol	1211	caraway	+			
aldehydes						
Acetaldehyde	420	aldehydic	+	+	+	+
Propanal	491	acetaldehydic	+		+	+
1-methylpropanal	517	aldehydic	+			
2-methylpropanal	519	aldehydic	+	+		
Butanal	565	chocolate		+	+	+
But-(E)-2-enal	665	floral	+	+		+
2-methylpentanal	763	cheeese			+	
Furfural	844	almond			+	
5-methylfurfural	952	acidic	+			
Benzaldehyde	968	almond	+			
terpenes						
Alpha-Pinene	950	camphor		+		
Beta-pinene	958	dry			+	
Alpha-Phalladrene	1002	citrus		+		
p-Cymene	1029	aromatic	+			
1,8-cineole	1048	camphor			+	
Gamma-Terpinene	1081	citrus; etheral	+	+	+	
esters						
Methyl formate	387	agereeable; fruity	+	+	+	+
Isopropyl acetate	652	banana	+			+
Methyl isobutyrate	655	apple			+	
Methyl butanoate	735	apple	+		+	
Methyl hexanoate	933	acetone		+	+	
hydrocarbons						
Pentane	478	alkane			+	+
Heptane	705	alkane		+		+
1-hydroxy-2(methylthio)-ethane	848	meaty			+	
N-compounds						
Pyrolle	754	chloroform		+	+	+
Pyridine	755	cold meat fat	+	+		
2-acetyl-1-pyrroline	926	meaty	+			
acids						
pentanoic acid	905	acidic		+	+	
Hexanoic acid	1004	cheese			+	
C-compounds						
Dihydro-2(3H)-Furanone	811	aromatic	+			
S-compounds						
Dimethyl trisulfide	970	aliaceous	+		+	
pyrazines						
Pyrazine	725	bitter	+			+
thiols						
Heptyl mercaptan	1027	onion		+	+	

^1^ DB5–Order of elution in DB-5 non-polar column; ^2^ NC—negative control (pork loins soaked in nitrite-free brine); PC—positive control (pork loins soaked in brine containing sodium nitrite); B1—pork loins soaked in brine containing plasma-activated powder of cow milk; B2—pork loins soaked in brine containing plasma-activated powder of soy milk; ^3^ the “+” sign indicates the presence of a compound.

## Data Availability

The data presented in this study are available on request from the corresponding author.
